# Dedifferentiated chondrosarcoma with leukocytosis and elevation of serum G-CSF. A case report

**DOI:** 10.1186/1477-7819-4-37

**Published:** 2006-07-04

**Authors:** Akio Sakamoto, Hidetaka Yamamoto, Kazuhiro Tanaka, Shuichi Matsuda, Katsumi Harimaya, Yoshinao Oda, Masazumi Tsuneyoshi, Yukihide Iwamoto

**Affiliations:** 1 Department of Orthopaedic Surgery, Graduate School of Medical Sciences, Kyushu University, Fukuoka, 812-8582, Japan; 2 Department of Anatomic Pathology, Graduate School of Medical Sciences, Kyushu University, Fukuoka, 812-8582, Japan

## Abstract

**Background:**

G-CSF is known to function as a hematopoietic growth factor and it is known to be responsible for leukocytosis. G-CSF-producing tumors associated with leukocytosis include various types of malignancies.

**Case presentation:**

We report the case of a 72-year-old man with dedifferentiated chondrosarcoma characterized by dedifferentiated components of malignant fibrous histiocytoma- or osteosarcoma-like features in addition to conventional chondrosarcoma, arising from his pelvic bone. After hemipelvectomy, when local recurrence and metastasis were identified, leukocytosis appeared and an elevated level of serum granulocyte-colony-stimulating factor (G-CSF) was also recognized. The patient died of multiple organ failure 2 months after surgery. Autopsy specimens showed that the histological specimens of the recurrence and metastasis were dedifferentiated components, without any conventional chondrosarcoma components. G-CSF was expressed only in the dedifferentiated components, not in the chondrosarcoma components, immunohistochemically.

**Conclusion:**

This is the first report of chondrosarcoma, or any other primary bone tumor, with leukocytosis, probably stimulated by tumor-produced G-CSF from the dedifferentiated components.

## Background

Granulocyte-colony stimulating factor (G-CSF) enhances differentiation along the neutrophil lineage, and accelerates maturation of metamyelocytes into mature neutrophils. G-CSF also prolongs the survival of neutrophils and their precursors, including stem cells[1]. Consequently, G-CSF is known to function as a hematopoietic growth factor and it is known to be responsible for leukocytosis. Normally, the serum G-CSF level is very low [2]. Production of granulocyte colony-stimulating factor (G-CSF) by tumor cells was first identified in lung carcinoma in 1977 [3]. G-CSF-producing tumors associated with leukocytosis include various types of malignancies, including lung caner [4,5], colon cancer [4], stomach cancer [4], thyroid caner [6], cervical cancer [7], malignant fibrous histiocytoma of soft tissue [8].

Dedifferentiated chondrosarcoma accounts for approximately 10% of all chondrosarcomas [9]. Dedifferentiated chondrosarcoma shows both a rapid growth rate and metastatic spread, and it has a significantly worse survival rate compared with conventional chondrosarcoma [10,11]. Dedifferentiated chondrosarcoma shows both a rapid growth rate and metastatic spread, and it has a significantly worse survival rate compared with conventional chondrosarcoma. The overall 5-year survival rate in conventional chondrosarcoma patients varies between 48%-60%, on the other hand, dedifferentiated chondrosarcoma is highly lethal, with less than a 10% survival rate after 1 year, with practically no long-term survivors [9]. Dedifferentiated chondrosarcoma is characterized by the coexistence of conventional chondrosarcoma and dedifferentiated components. The dedifferentiated components can features of malignant fibrous histiocytoma (MFH), osteosarcoma, angiosarcoma, fibrosarcoma, rhabdomyosarcoma, leiomyosarcoma and giant cell tumor [11]. We report a case of dedifferentiated chondrosarcoma with leukocytosis, probably due to stimulation by tumor-produced G-CSF. This chondrosarcoma with leukocytosis and an elevated serum G-CSF level is the first such report not only of chondrosarcoma, but also of any such primary bone tumor with leukocytosis.

## Case presentation

**Figure 1:**
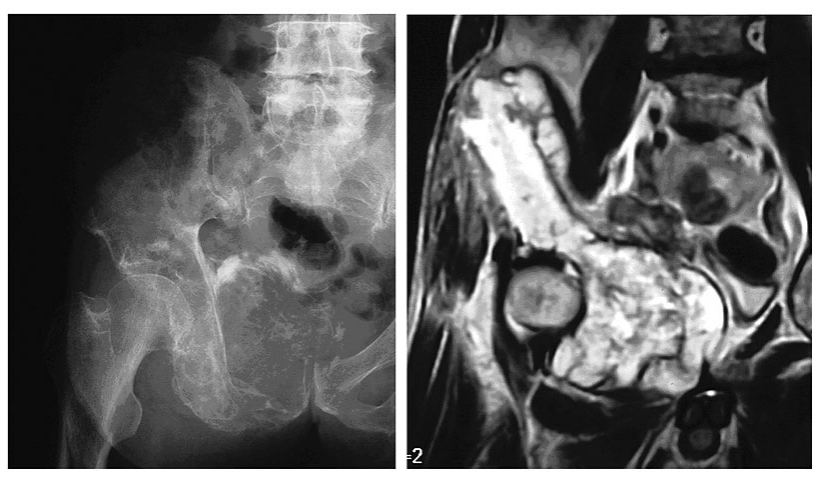
Roentgenographs show an expansive lesion with punctate calcification in the right pelvic bone (left). MRI shows a lobulated lesion, protuberant to the pelvic cavity, in the pelvic bone with high-intensity on T2-weighted image (right).

**Figure 2:**
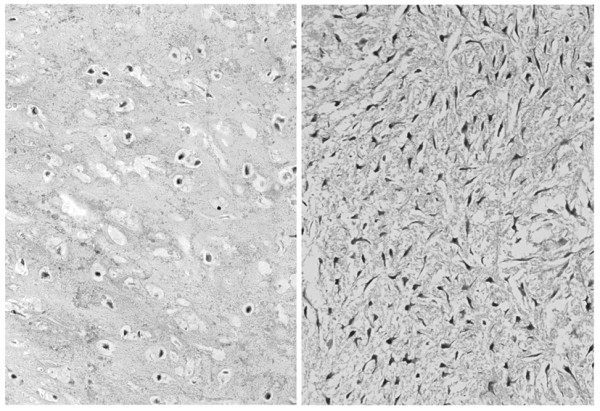
Dedifferentiated chondrosarcoma; well-differentiated components show conventional chondrosarcoma composed of atypical chondrocytes with hyaline cartilage matrix (left) and with myxoid matrix (right) (Hematoxylin and Eosin original magnification × 180).

**Figure 3:**
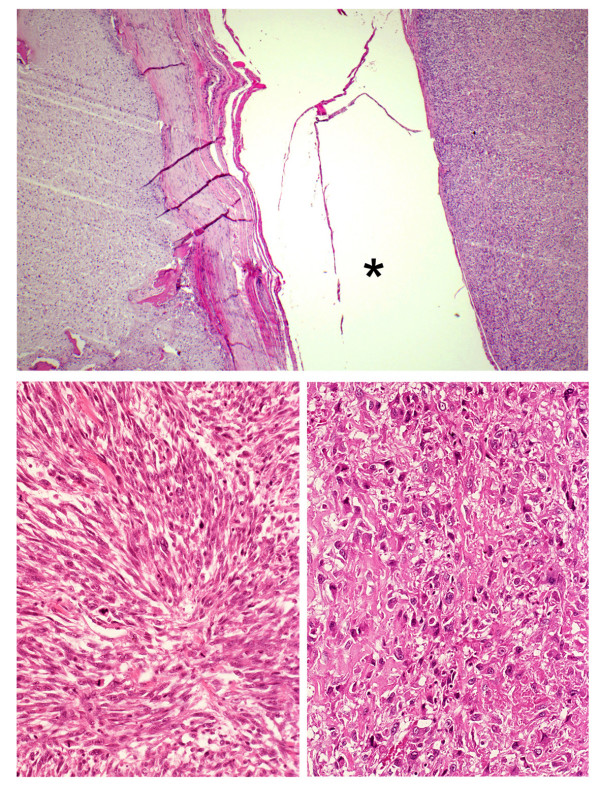
Dedifferentiated chondrosarcoma is characterized by the coexistence of conventional chondrosarcoma (top; left side) and dedifferentiated components (top; right side). Dedifferentiated components are composed of atypical spindle cells in a storiform pattern, resembling MFH (bottom; left), or osteosarcoma with lace-like osteoid formation (bottom; right). Asterisk (*) shows artificial space. (Hematoxylin and Eosin original magnification top; × 50, bottom; × 180).

A 72-year-old man, who had been suffering from right hip pain for 7 years when walking, was referred to our institute. Roentogenography of the right pelvic bone showed an expansion of the cortical contour and a soft-tissue mass with punctate calcification (figure 1). Magnetic resonance imaging (MRI) showed a lobulated lesion expanding over the entire right pelvic bone with low-intensity on T1-weighted imaging and iso- to high-intensity on T2-weighted imaging, with the tumor protuberant to the pelvic cavity (figure 1). Right hemipelvectomy was carried out. Histological sections showed a conventional chondrosarcoma having atypical chondrocytes with hyaline cartilage matrix or myxoid matrix (figure 2). In a small section, dedifferentiated components were also identified, and these dedifferentiated components consisted of atypical spindle cells arranged in short fascicles or a storiform pattern showing MFH-like features, plus some pleomorphic cells with lace-like osteoid formation showing osteosarcoma-like features (figure 3). Therefore, the final diagnosis was dedifferentiated chondrosarcoma because of the coexistence of conventional chondrosarcoma and dedifferentiated components.

**Figure 4:**
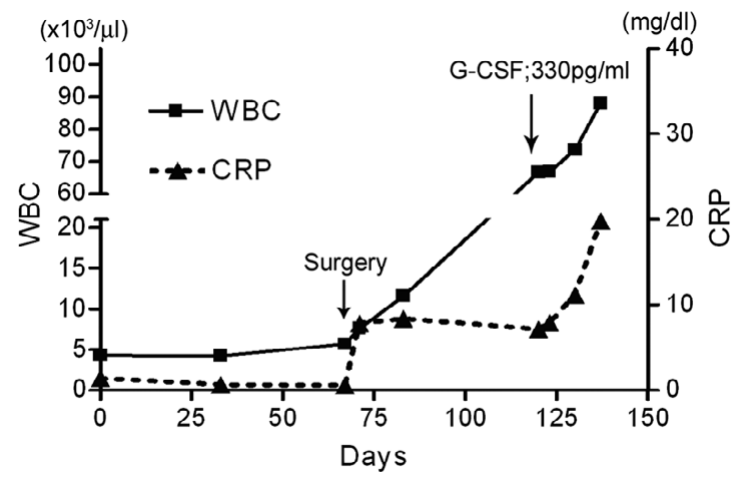
Leukocytosis appeared after the surgery. Elevated serum G-CSF level is shown (arrow). C-reactive protein is also increased.

**Figure 5:**
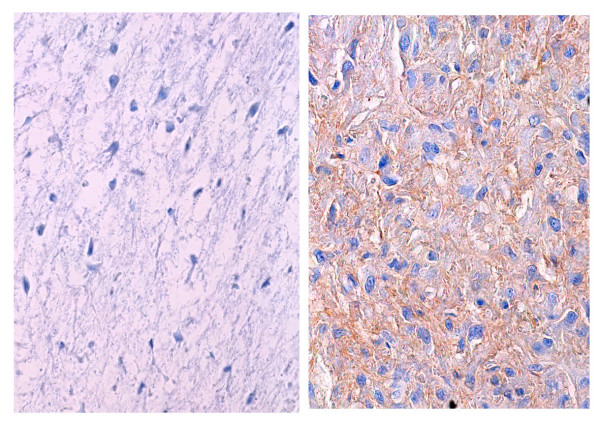
Immunohistochemistry of G-CSF is absent in the conventional chondrosarcoma components (left), while is seen in the dedifferentiated components (right) (Immunohistochemistry original magnification × 250).

Preoperative laboratory data were not remarkable. After surgery, the laboratory data also showed leukocytosis predominantly in the neutrophils with an elevated level of C-reactive protein (figure 4). The serum level of G-CSF was also elevated (330 pg/ml [normal, <8 pg/ml]). Magnetic resonance imaging (MRI) and computed tomography (CT) revealed evidence of local recurrence and metastatic lesions in the lungs. Flow-cytometry indicated no evidence of leukemia and serological studies showed no evidence of specific infections, such as candidiasis or tuberculosis. Despite the administration of several antibiotics, the leukocytosis did not disappear. Two months after the surgery, the patient died of multiple organ failure. An autopsy was carried out, and in addition to the lung metastasis, metastasis was also found in the liver, thyroid, diaphragm, adrenal gland, digestive tract and skin. The histology of the recurrent and metastatic lesions was not conventional chondrosarcoma but only dedifferentiated components. Immunoexpression of G-CSF (anti-G-CSF [Ab1], Calbiochem, San Diego CA, USA) was seen in the dedifferentiated components, but not in the conventional chondrosarcoma components (figure 5).

## Discussion

In the current report, we present a case of dedifferentiated chondrosarcoma with leukocytosis and an increased serum G-CSF level. In a previous report, serum G-CSF was not found to be increased in bone tumors, including osteosarcoma, chondrosarcoma, Ewing/PNET and giant cell tumor, compared to the control [12]. Therefore, an elevation of serum G-CSF seemed to be a specific finding in the current case. Leukocytosis in the current case was probably stimulated by tumor-produced G-CSF. G-CSF-producing tumors associated with leukocytosis are a well-known phenomenon. Besides tumor cells themselves, neoplastic necrosis produces multiple cytokines, including G-CSF, and causes leukecytosis [15]. However, there is less necrotic area in this dedifferentiated chondrosarcoma. Moreover, the degree of leukocytosis associated with surgery, such as reactive local inflammation and healing wound, is mild, which is different from leukocytosis caused by tumor-produced G-CSF. Taken together, the leukocytosis seemed to be strongly associated with an elevated serum G-CSF of neoplastic origin. Secondary non-epithelial tumors with leukocytosis have been reported, among which there was a case of undifferentiated sarcoma following a fracture [13] and a case of angiosarcoma following burns [14]. Among the primary bone and soft-tissue tumors, only 5 cases of soft-tissue tumor have been reported, among which there was one case of MFH [8] and 4 cases of liposarcoma [15-18]. However, to our knowledge, no bone tumor with leukocytosis plus an elevated serum G-CSF level has been reported.

In the current case, leukocytosis appeared after the surgery, when recurrence and metastasis also appeared. The histology of the recurrent and metastatic lesions was not conventional chondrosarcoma but only dedifferentiated components. Therefore, it would seem that the G-CSF production could have been associated with the dedifferentiated components. Supporting this idea, G-CSF expression was found only in the dedifferentiated components, and not in the conventional chondrosarcoma components, immunohistochemically. As described, the histology of G-CSF-producing soft-tissue tumors was MFH and liposarcoma [8,15-18]. The subtype of these reported liposarcoma cases was pleomorphic type [15,16] and dedifferentiated type [17,18], in 2 cases each. G-CSF production in bone and soft-tissue tumors may be related with undifferentiated sarcoma, such as MFH or sarcomas of pleomorphic type or dedifferentiated type.

The current case of dedifferentiated chondrosarcoma had a rather long history of 7 years without leukocytosis prior to the initial surgery. The resected specimens had conventional chondrosarcoma components in most part, and dedifferentiated components only in a small part. It is not clear why there was no leukocytosis before the surgery, even though there were dedifferentiated components. One theory is that the volume of dedifferentiated components with G-CSF production was too small to cause leukocytosis. Another theory is that there was no production at the beginning, and dedifferentiated components acquired the function of G-CSF production during the course of tumor development. When leukocytosis appeared, the tumor had already recurred and metastasis had developed, and the patient died 2 months after the surgery. G-CSF has been shown to induce tumor cell proliferation [19-21]. In addition, in a previous report, tumor-related leukocytosis is linked with poor prognosis in patients with lung carcinoma. Patients with lung carcinoma with leukocytosis had a poor outcome compared with the other patients without leukocytosis [5]. In the same line, G-CSF may have contributed to the aggressive behavior of dedifferentiated chondrosarcoma in the current case.

It is uncertain whether dedifferentiated chondrosarcoma consists primarily of two cell types or whether dedifferentiation develops secondarily. Molecular genetic study of one case has shown a substantial number of genetic alterations, as well as severe aneuploidy and loss of heterozygosity in both the components of dedifferentiated chondrosarcoma [22]. LOH (loss of heterozygosity) in Rb predominantly occurs in the dedifferentiated components of dedifferentiated chondrosarcomas [23]. Gene alterations in *p53*, *Rb *and *FHIT *are reported to be responsible for the "switch" to a high-grade anaplastic sarcoma (dedifferentiated components) from monoclonal origin of demonstrated dedifferentiated chondrosaroma [24]. It is also possible that the function of G-CSF production was acquired as a result of genetic alteration during the course of tumor development in the current case.

## Conclusion

In this report, we present a case of dedifferentiated chondrosarcoma with leukocytosis. The leukocytosis seemed to be associated with an elevated serum G-CSF level, which may be related to dedifferentiated components in the current dedifferentiated chondrosarcoma case.

## Competing interests

The author(s) declare that they have no competing interests.

## Authors' contributions

**AS **drafted the manuscript and carried out the immunohistochemical studies. **HY **and **YO **carried out the immunohistochemical studies. **KT**, **SM **and **KH **participated in the design of the study and performed. **MT **and **YI **conceived of the study, and participated in its design and coordination and helped to draft the manuscript. All authors read and approved the final manuscript.
